# Eplerenone Reverses Cardiac Fibrosis via the Suppression of Tregs by Inhibition of Kv1.3 Channel

**DOI:** 10.3389/fphys.2018.00899

**Published:** 2018-07-13

**Authors:** Pei-Pei Shao, Chang-Jiang Liu, Qi Xu, Bo Zhang, Shao-Hua Li, Yang Wu, Zhan Sun, Lu-Feng Cheng

**Affiliations:** ^1^Department of Pharmacology, School of Pharmacy, Xinjiang Medical University, Ürümqi, China; ^2^Department of Immunology, School of Pre-clinical Medicine, Xinjiang Medical University, Ürümqi, China; ^3^Key Laboratory of Xinjiang Phytomedicine Resource and Utilization, Ministry of Education, Shihezi University, Shihezi, China; ^4^Center of Functional Experiment, School of Pre-clinical Medicine, Xinjiang Medical University, Ürümqi, China

**Keywords:** eplerenone, cardiac fibrosis, CD4^+^CD25^+^ Treg lymphocytes, Kv1.3 channel, TGF-β

## Abstract

**Background:** Fibroblast proliferation is a critical feature during heart failure development. Previous studies reported regulatory T-lymphocytes (Tregs)’ protective role against myocardial fibrosis. However, notably, Tregs also secrete fibrogenic cytokine TGF-β when activated. This study aimed to clarify the intriguing link between Tregs and fibrosis, the role of Tregs Kv1.3 potassium channel (regulating T-lymphocytes activation) in the fibrosis process, and how selective aldosterone receptor antagonist Eplerenone affects Tregs and fibrosis through its action on Kv1.3 channel.

**Methods and Results:** After co-incubation with Tregs, cardiac fibroblast proliferation (CCK-8 assay) and levels of collagen I, III, and Matrix metalloproteinase2 (ELISA) significantly elevated. Cell viability assays, Kv1.3 channel mRNA (RT-qPCR), and protein expression (In-Cell Western Blotting) revealed Tregs were activated/proliferated when co-cultured with fibroblasts. Treg intracellular TGF-β level increased by 5.8-fold, far more than that of intracellular IL-10, extracellular TGF-β and IL-10 (ELISA). And 30 μM eplerenone suppressed Tregs proliferation by 82.77% and furthermore, suppressed intracellular TGF-β level to a significantly greater extent than that of intracellular IL-10, extracellular TGF-β and IL-10. Moreover, the Kv1.3 current (whole-cell patch clamp) of Tregs in congestive heart failure patients and rats (induced by coronary artery ligation and exhaustive exercise) elevated by >4-fold than that of healthy volunteers and control rats, whereas 30 μM eplerenone suppressed the current by >60% in control Tregs. In addition, docking calculations (AutoDock software 4.0 suite) showed eplerenone has higher H-bond energy with Kv1.3 channel than other selective blockers.

**Conclusion:** Immuno-regulation in the late stage of CHF activates Tregs proliferation via the upregulation of Kv1.3 channels, which promotes cardiac fibrosis by primarily secreting TGF-β. Taken together, eplerenone’s high affinity to Kv1.3 channel enables it to antagonize the Kv1.3 channels directly to suppress Tregs proliferation, which in turn may play an immuno-regulatory role during CHF.

## Introduction

Eplerenone, a highly selective aldosterone receptor antagonist used to treat heart failure, is known to reduce all-cause mortality and sudden cardiac death when used in combination with angiotensin converting enzyme inhibitors and β-blockers (Seferovic et al., 2015; Heggermont et al., 2016; Ponikowski et al., 2016).

The role of inflammation in the development of cardiovascular diseases has been investigated extensively (Borthwick et al., 2013; Prabhu and Frangogiannis, 2016). Local myocardial injury and cardiomyocyte necrosis, i.e., ischemia, atherosclerosis, and myocardial infarction (MI) trigger intense inflammatory responses. However, dysregulated chronic inflammatory response may result in pathological wound repair, accumulation of permanent fibrotic scar tissue at the site of injury, failure of the tissue to regain normal function, and lead to myocardial fibrosis eventually.

A variety of immune cells and cytokines have been reported to play a direct or indirect role in the pathogenesis of myocardial fibrosis. Th17 cells play a vital role in the development of autoimmune disease and anaphylactic reactions, while CD4^+^CD25^+^ regulatory T lymphocytes (Treg cells) have anti-inflammatory activity and maintain immune homeostasis by secreting anti-inflammatory cytokines, i.e., interleukin (IL)-10 and transforming growth factor (TGF)-β. Therefore, the balance between Th17 and Treg cells is vital to the development/prevention of inflammation and autoimmune diseases (Sun et al., 2017).

Several studies have shown that Tregs are inversely correlated to cardiovascular diseases (Tang et al., 2010; Hofmann and Frantz, 2013; Frieler and Mortensen, 2015). Accordingly, Tregs have been established as a valuable prognostic marker and a therapeutic target in the treatment of heart failure (Meng X. et al., 2016). However, the exact role of Tregs in organ fibrosis under inflammatory conditions remains controversial. Some hypothesized that immune-suppressing Tregs play a profibrotic role, and suppression of TGF-β can alleviate fibrosis of the heart, liver, and kidneys (Wei, 2011). In the contrary, Cao et al. (2013) suggested that intravenous Tregs can reverse myocardial fibrosis mediated by the secretion of IL-10. A number of *in vivo* studies have shown that the suppression of TGF-β can alleviate fibrosis of the heart, liver, and kidneys in various animal species (Borthwick et al., 2013; Meng X.M. et al., 2016). The voltage-gated Kv1.3 channel, a T lymphocyte-specific ion channel, is involved in the differentiation and activation of T lymphocytes, and has been validated as a therapeutic target for the treatment of diversified autoimmune diseases (Gilhar et al., 2016; Wang et al., 2016; Chandy and Norton, 2017). Many studies proposed that Kv1.3 channels affect proliferation through K^+^ efflux and membrane hyperpolarization, which promotes Ca^2+^ influx to activate Ca^2+^-dependent transcriptional factors (Wang and Xiang, 2013; Orban et al., 2014; Perez-Garcia et al., 2018).

This study was to investigate the involvement of Tregs in the development of cardiac fibrosis as well as to determine whether eplerenone influences the activation and/or proliferation of Tregs in the treatment of congestive heart failure (CHF).

## Materials and Methods

### Reagents

Fetal bovine serum (FBS) and RPMI1640 were purchased from Thermo Fisher Scientific (Waltham, MA, United States), HISTOPAQUE^®^-1077/1083 from Sigma-Aldrich (St. Louis, MO, United States), and the primary mouse anti-rat KCNA3 and β-actin antibodies from Abcam (Cambridge, MA, United States). (PerCP-CyTM5.5) Mouse Anti-Human CD3, (FITC) Mouse Anti-Human CD4, (APC) Mouse Anti-Human CD183 and (PE) Mouse Anti-Human CD196; (FITC) Mouse Anti-Human CD4, (APC) Mouse Anti-Human CD25 and (PE) Mouse Anti-Human CD127 for flow cytometry measurement of Th17 and Tregs were purchased from Becton Dickinson (Franklin lake, NJ, United States). IRDye^®^800CW goat anti-mouse antibody was purchased from LI-COR Biosciences (Fullerton, CA, United States), and Biotin-conjugated mouse anti-rat CD4 as well as PE-conjugated mouse anti-rat CD25 were purchased from Becton Dickinson (Franklin Lake, NJ, United States). Biotin-conjugated mouse anti-human CD4, PE-conjugated mouse anti-human CD25 Multisort MicroBeads, LS columns and MiniMACS^TM^ separator were purchased from Miltenyi Biotec (Bergisch Gladbach, Germany). SYBR^TM^ Select Master Mix and Trizol were purchased from Life Technologies (Waltham, MA, United States). RevertAid First Strand cDNA Synthesis Kit was purchased from Thermo scientific (925 West 1800 South Logan, United States). Kv1.3, KCa3.1, CRAC gene sequences were purchased by Quintarabio (Ürümqi, Xinjiang, China). The CCK-8 Kit was purchased from Bosterbio (Pleasanton, CA, United States). ELISA assays for Collagen I, Collagen III and Matrix metalloproteinase2 (MMP2), brain natriuretic peptide (BNP), IL-1β, IL-6, IL-17, IFN-γ, and TNF-α were purchased from CUSABIO Biotech (Wuhan, Hubei, China), while those for TGF-β, and IL-10 as well as the Masson Stain Kit were purchased from Jiancheng Bioengineering Institute (Nanjing, Jiangsu, China). Psora-4 and eplerenone were purchased from Sigma-Aldrich (St. Louis, MO, United States).

### CHF Patients Inclusion/Exclusion Statements

Twenty-five patients with chronic heart failure (CHF) aged 30–80 years old were collected from Department of Heart Failure, the First Affiliated Hospital of Xinjiang Medical University from January 2016 to May 2017. The experiment was approved by the Ethics Committee of the First Affiliated Hospital. The clinical general data were collected. Cardiac function was measured by echocardiography to evaluate left ventricular systolic/diastolic function, and the immuno-factors were determined by flow cytometry and/or ELISA. 15 healthy volunteers aged 30–55 years old were screened from the Health Examination Center of the First Affiliated Hospital as the control group.

CHF patients inclusion criteria: (1) voluntary informed participation with no limitation of gender; (2) according to the diagnostic criteria, the cardiac function was at NYHA grade III–IV; (3) left ventricular ejection fraction (LVEF) ≤ 40%; (4) 3000 ≤ Brain Natriuretic Peptide (BNP) ≤ 5000.

Exclusion criteria: (1) acute or chronic infection; (2) autoimmune diseases; rheumatic diseases with rheumatic activity within 3 months; recent use of drugs affecting immune response (e.g., corticosteroids); (3) acute cardiovascular diseases and organic heart diseases within 3 months, e.g., acute pulmonary edema, acute left ventricular dysfunction, unstable angina pectoris, acute myocardial infarction as well; (4) others: e.g., abnormal thyroid function; obvious hepatic and renal dysfunction; malignant tumor; pregnancy.

### Animal Ethics

Male Sprague–Dawley rats were purchased from the Animal Experimental Center (Xinjiang Medical University). All rats weighed between 180 and 220 g at the time of the experiments. All procedures involving animals were performed under an NIH Guidelines approved protocol in accordance with the Institutional Animal Care and Use Committee (approved by AAALAC in England) at the First Affiliated Hospital of Xinjiang Medical University. Rats were anesthetized with a 2% isoflurane gas mixture of oxygen inhalation for 3–5 min using a Matrix VIP3000 Isoflurane Vaporizer (Matrix, New York, United States) during coronary artery ligation (CAL) surgery, echocardiographic and hemodynamic detection, and blood and major organs were isolated for further detection and sample preparation. The fully anesthesia was confirmed with no reflex response to foot clamp. Neonatal rats were euthanized via cervical dislocation, and hearts were isolated for cardiac fibroblasts enrichment. All surgeries and follow-up analyses complied with blind principle.

### Establishment of a Rat Model of CHF

Congestive heart failure was initiated with coronary artery ligation on rats according to Ren et al. (2017). Briefly, after being anesthetized, the left coronary artery was ligated with a 7-0 suture near the initiation of coronary artery (the point between left auricle inferior margin and pulmonary conus). The myocardial infarction was confirmed with a pale surface color and S-T segment elevation by electrocardiographic (ECG) monitoring. To further establish the CHF model, 30-min-per-day exhausted swimming was implemented on every surgery rats sustaining for 15 days. 10–12 weeks later after coronary ligation, when the indexes of B-ultrasound reached the following criteria (Ponikowski et al., 2016): left ventricular ejection fraction (LVEF) <50% and/or a 40% suppressant in the maximum systolic velocity of left ventricular pressure (LV + d*p*/d*t*_max_), the following-up experiments would be performed.

### Isolation of CD4^+^CD25^+^ Tregs

CD4^+^CD25^+^ Tregs from human peripheral blood were isolated in accordance with the manufacturer’s instructions. Briefly, mononuclear cells precipitation was separated by HISTOPAQUE^®^-1077 human lymphocytes separation medium and centrifugation (1600 rpm, 20 min, 4°C). CD4^+^ T cells were then isolated by negative selection using Biotin-Antibody Cocktail with Multisort MicroBeads and Anti-Biotin MicroBeads (Miltenyi Biotec, GER) at 4°C, passing through the LS separate column around the MiniMACS separator, releasing and terminating by Multisort Release and Stop Reagents sequentially at 4°C. CD4^+^ T cells were enriched and CD4^+^CD25^+^ T cells were further screened for positive expression of CD25 using CD25 MicroBeads II human with MicroBeads and MiniMACS separator. The purity of isolated CD4^+^CD25^+^ Tregs was greater than 95%, and incubated with 10% FBS (Gibco, United States) media at 37°C in a 5% CO_2_ incubator.

The single cell suspensions from spleen of Sprague–Dawley rat were prepared by passing the sheared tissue through a 200-mesh stainless steel strainer (Sigma-Aldrich, St. Louis, MO, United States), and following the manufacturer’s instructions, mononuclear cells precipitation was separated by HISTOPAQUE^®^-1083 rat lymphocytes separation medium and centrifugation (1600 rpm, 20 min, 4°C). CD4^+^ T cells were then isolated by positive selection using Biotin-Mouse Anti-Rat with Multisort MicroBeads and Anti-Biotin MicroBeads (Miltenyi Biotec, GER) at 4°C, passing through the LS separate column around the MiniMACS separator, releasing and terminating by Multisort Release and Stop Reagents sequentially at 4°C. CD4^+^ T cells were enriched and CD4^+^CD25^+^ T cells were further screened for positive expression of CD25 using PE-Mouse Anti-Rat CD25 with MicroBeads and MiniMACS separator. The purity of isolated CD4^+^CD25^+^Tregs was greater than 95%, and incubated with 10% FBS (Gibco, United States) media at 37°C in a 5% CO_2_ incubator.

### Primary Culture of Cardiac Fibroblasts of Rats

Cardiac fibroblasts (CFs) were isolated from hearts of neonatal (3-day-old) rats by digestion and differential adhesion, as described previously (Barry et al., 2013). Briefly, the heart was removed, chopped into tiny pieces, and digested in phosphate-buffered saline (PBS) solution containing 0.25% trypsin on a shaking platform (30 rpm) at 4°C overnight. The digested tissue was then treated with 0.1% collagenase II for 8–10 min with 3–4 times at 37°C. The digestion was discontinued along with 10% FBS. After centrifugation (1300 rpm, 5 min, 20°C), the resulting supernatant was discarded and the precipitated cells were re-suspended and seeded in 50 mL flasks before being cultured for 90 min at 37°C in an incubator (1st differential adhesion) and then 40 min (the 2nd differential adhesion). Bromodeoxyuridine (BrdU: 0.1 mM) was then added to terminate the growth of the remaining fibroblasts. Cells (5 × 10^5^ per well) were planted into plates with media containing 10 g/L FCS, 1 g/L double antibody, 1 g/L glutamate, and 1 g/L non-essential amino acid was replaced every 48 h. The cells were purified to obtain the primary fibroblasts, and 3–4 generations were collected for subsequent experiments.

### Co-culture of Cardiac Fibroblasts and Tregs *in Vitro*

The treatment protocol included the following groups: CFs, Tregs, CFs + EPL, Tregs + EPL, CFs + Tregs, and CFs + Tregs + EPL. Fibroblasts were planted into 96-well plates with 10^4^ cells/hole at 37°C in a 5% CO_2_ incubator for 48 h. When an adhesion rate of 80.0 ± 5.0% was achieved, the fibroblasts were incubated with PBS for 12 h. In the meantime, the Tregs were treated in the same way. Next, the fibroblasts and Tregs were mixed and treated with eplerenone in RPMI 1640 containing 1 g/L double antibody for 48 h. The Tregs were replaced with fresh ones after 24 h due to the viability being reduced by this time.

Since cardiac fibroblasts are passaged cells while Tregs are not, for different *in vitro* experiments, fibroblasts were extracted from same batch of newborn rats (*n* = 10) and Tregs from different batches of adult rats.

### Flow Cytometric Analysis of Th17 and Treg Cells

Two hundred microliter anticoagulant total blood mixed with 5 μL (PerCP-CyTM5.5) Mouse Anti-Human CD3, (FITC) Mouse Anti-Human CD4, (APC) Mouse Anti-Human CD183 and (PE) Mouse Anti-Human CD196 for Th17 cells or with 5 μL (FITC) Mouse Anti-Human CD4, (APC) Mouse Anti-Human CD25 and (PE) Mouse Anti-Human CD127 for Treg cells were reacted away from light for 30 min, followed by adding lytic erythrocyte, centrifuging (1000 rpm, 5 min, 20°C) and washing cells for three times. Lastly 300 μL buffer solution was fully mixed the cells for flow cytometer measurement.

### Echocardiography

Echocardiography was performed with an ultrasound instrument (HP 5500, United States) on day 70 post-surgery. After being anesthetized, the anatomical data of the rat heart were obtained by averaging results from three consecutive heart beats. On day 4 after ultrasound assessment, rats underwent right carotid artery catheterization into the left ventricle and using a biological experimental system (Powerlab, Australian); hemodynamic indexes were recorded after steady state was achieved.

### Assessment of Cardiac Fibrosis by Masson-Trichrome Staining

A horizontal section of left ventricle stained with Masson-trichrome was examined to evaluate the infarct size (cardiac fibrosis). Mice with infarct size less than 25% of total area of left ventricle were excluded.

### Cell Viability Assay

The CCK-8 Kit was employed to analyze cell proliferation rates. Due to the non-adherent nature of the Tregs, the cells were re-suspended by gentle pipetting up and down to ensure that both cell types were accurately represented. Tregs were collected after 24 and 48 h, washed, and seeded into 96-well plates for subsequent analysis. Both cell types were incubated for an additional 2 h at 37°C. CCK-8 (10 μL) was added to each well and the volume in each well was made up to 200 μL with PBS. Absorbance (OD value) was detected at a double-wavelength of 450 nm and 610 nm using a microplate reader (Thermo Multiskan Spectrum, Waltham, MA, United States).

### Analysis of Kv1.3 Channel on Tregs Using the Whole-Cell Patch-Clamp Technique

Tregs were “floated off” in the working chambers of culture plates for 10 min before being washed and then studied within 20–90 min after plating in the whole-cell patch clamp technique with an EPC-10^+^ HEKA amplifier (Germany). Patch pipettes were pulled from micropipette raw glass (OD 1.5 mm × ID 0.84 mm, Vital Sense Scientific Instrument, China) to resistances of ∼5 MΩ when submerged in the bath solution. The pipette solution contained 150.0 mM KCl, 1.0 mM CaCl_2_, 1.0 mM MgCl_2_, 10.0 mM HEPES, and 10.0 mM K_2_EGTA (pH 7.20, adjusted with KOH). The perfusing solution contained 150.0 mM NaCl, 4.5 mM KCl, 1.0 mM CaCl_2_, 1.0 mM MgCl_2_, and 10.0 mM HEPES (pH 7.35, adjusted by NaOH), and Kv1.3 currents were elicited with voltage ramps from -80 to +40 mV of 300-ms duration applied every 30 s, as described previously (Zhao et al., 2014; Zhang et al., 2016). Fast and slow capacitances were compensated before every recording. Cell capacitance and access resistance were continuously monitored during recordings. Kv1.3 current densities were determined by the cell capacitance.

### mRNA Expression of Ion Channels in Tregs by RT-qPCR

Total RNA was extracted from the Tregs harvested from every treatment group, after which reverse transcription was immediately carried out as described in the manufacturer’s instructions. PCR amplification was performed using the Green Master Mix Kit (Life Technologies, Waltham, MA, United States), and the reagents, prepared on ice, consisted of the following: 6.4 μL H_2_O, 10 μL SYBR, 0.8 μL each primer (forward and reverse), and 2.0 μL sample. The PCR steps were as follows: 50.0°C for 2 min; 95.0°C for 2 min; 95.0°C for 15 s with 40 loops; 60°C for 15 s; and 95°C for 15 s. Primer sequences are shown in **Table 1**.

**Table 1:** Gene sequences of ion channels.

Gene	Gene ID	Upstream (5′–3′)	Downstream (5′–3′)
G3PD	GAPDH	GGCAGCCTGTTGGAAAAGAA	GGCAGCCTGTTGGAAAAGAA
Kv1.3	KCNA3	GGCAGCCTGTTGGAAAAGAA	GGCAGCCTGTTGGAAAAGAA
KCa3.1	KCNN4	ACTGGAGTCATGGGTGTCTG	ATGAGACTCCTTCCTGCGAG
CRAC	CRACR2A	TCTCCGTTGAAGAAGACCCC	GGCAGCCTGTTGGAAAAGAA


### In-Cell Western Blotting Assay of Kv1.3 Channel on Tregs

A Tregs suspension was seeded into 96-well plates with a “U”-type black wall and the cells were fixed with 4% paraformaldehyde. Cells were then sealed with TBS sealing liquid (prepared with 0.5% defatted milk powder) for an hour before being incubated with 50 μL KCNA3 primary antibody (diluted in 0.25 g/L defatted milk powder; 1:800) on a shaking platform (30 rpm) overnight at 4°C. After an hour of incubation with secondary antibody the following day, the resulting signals were detected using an Odyssey (LI-COR Biosciences, Fullerton, CA, United States) scanner with the following conditions: double channels of 700 and 800 nm, medium scanning quality, 169 μm dpi, 3.0 mm focal length, and level 5 of brightness.

### Measurement of BNP and Inflammatory Cytokine Levels by ELISA

Levels of BNP and inflammatory cytokines in the plasma were measured using commercially available ELISA Kits according to the manufacturer’s instructions. Data were analyzed using a microplate reader (Bio-Rad Coda, United States).

### Measurement of Extracellular and Intracellular TGF-β and IL-10 Levels in Tregs by ELISA

After 48 h of culture, post-centrifugal supernatant fluid was collected from each treatment group as extracellular fluid. The precipitated Tregs were subjected to repeated freezing and melting cycles to release the cell contents (intracellular fluid). The TGF-β and IL-10 levels in the extracellular and intracellular fluid samples were detected by ELISA Kits in accordance with the Kit instructions. Data were analyzed using a microplate reader (Thermo Multiskan Spectrum, United States).

### Expression of ECM-Associated Proteins by ELISA

After the CFs, CFs + Tregs, and CFs + Tregs + EPL cell groups were incubated for 48 h at 37°C in a 5% CO_2_ incubator, the culture medium was collected from each culture and assessed for secretion of Collagen I, Collagen III, and MMP-2 using ELISA method. Results were analyzed using a microplate reader (Bio-Rad Coda, United States).

### Structure Preparation and Molecular Docking

Atomic coordinates for the human renal potassium channel Kv1.3 structure, with accession number PDB ID: 4BGC (Bhuyan and Seal, 2015), were used. Initial atomic coordinates for all ligands were obtained from PubChem^1^. The molecular interactions between the Kv1.3 and the three ligands were computed using AutoDock 4.0 suite (Molecular Graphics Laboratory, La Jolla, CA, United States).

Docking calculations were carried out on the protein models. Essential hydrogen atoms, kollman charges, and solvation parameters were added with the aid of AutoDock tools. Affinity maps were calculated using probes corresponding to all possible atomic types present in the full set of ligands. Atom types were assigned by AutoDock Tools. Affinity maps of 86 × 72 × 110 A grid points and 0.375 A spacing were generated using the Autogrid program. AutoDock parameter set and distance-dependent dielectric functions were used in the calculation of the Van der Waals and the electrostatic terms, respectively. Docking simulations were performed using the Lamarckian genetic algorithm (LGA). The docking parameters set to perform each docking experiments was derived from 10 different runs. The best run coordinates of the compounds with enzyme were visualized and analyzed through AutoDock Tools for analysis of their mode of interaction with binding site residues.

### Statistical Analysis

ELISA response standard curve equations were obtained using Curve Expert 1.3 software (Biological Software, MN, United States Re seller), and patch clamp data were exported by Igor Pro 5.05A software (WaveMetrics Inc., Lake Oswego, OR, United States). Except for patch-clamp data (presented as mean ± SE), all data are presented as mean ± SD. One-way ANOVA and subsequent Student’s *t-*test (SPSS 22.0 statistical software, Chicago, IL, United States) were used to analyze comparative data, where differences with *P* < 0.05 indicated statistical significance.

## Results

### Immune System Alterations in CHF Patients

#### Th17/Treg Ratio in CHF Patients

**FIGURE 1:**
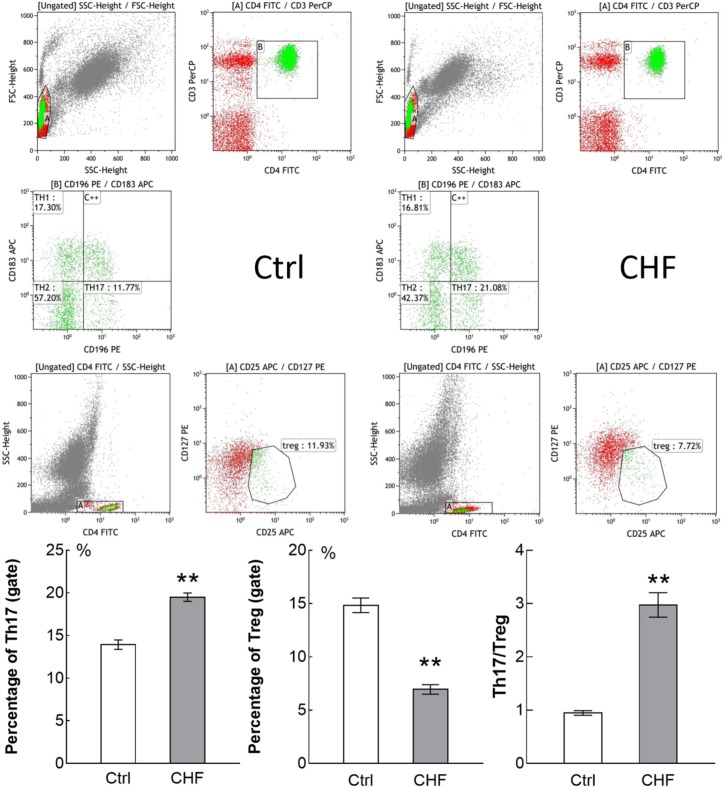
Flow cytometry assay showed the percentage of Th17 cells and Treg cells in CHF patients (25 cases) compared to control subjects (15 cases). The original flow cytometry plots of the two groups is displayed above, and histogram comparison of Th17, Treg cells and the ratio of Th17/Treg is displayed below. Th17 cells in CHF group was significantly higher than in control group (^∗∗^*P* < 0.01), while Treg cells in the CHF group was significantly lower than in control group (^∗∗^*P* < 0.01); thereby the ratio of Th17/Treg in the CHF group was significantly higher than that in the control group (by ∼3-fold, ^∗∗^*P* < 0.01).

Flowcytometry assay showed the quantity of Th17 cells in CHF patients was significantly higher than that in healthy people (19.50 ± 1.81 vs. 13.94 ± 1.55%, *n* = 25 vs. 15, ^∗∗^*P* < 0.01), while the quantity of Treg cells in the CHF was significantly lower than that in the control (6.95 ± 1.65 vs. 14.85 ± 1.92%, ^∗∗^*P* < 0.01) (**Figure 1**). Thereby the Th17/Treg ratio of the CHF patients was significantly higher than that of the healthy people (2.97 ± 0.86 vs. 0.95 ± 0.12, ^∗∗^*P* < 0.01).

#### Plasma IL-10 and TGF-β in CHF Patients

**FIGURE 2:**
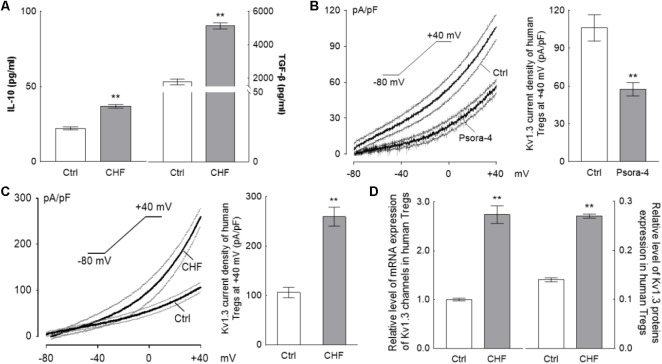
The changing of Tregs activation for CHF patients. **(A)** The histograms differences of serum IL-10 and TGF-β between the CHF patients and the control persons by ELISA assay. Levels of both IL-10 and TGF-β were elevated significantly in the CHF group compared to the blank control group, nevertheless, the elevation of IL-10 (65.9%) was far less than that of TGF-β (2.9-fold) (^∗∗^*P* < 0.01, *n* = 30 vs. 30). **(B)** The Kv1.3 channels on the Tregs membrane were identified by perfusion with 3 nM Psora-4 (a specific Kv1.3 channels inhibitor) under voltage ramp from –80 mV to +40 mV of 300 ms duration (*n* = 15, the black traces are averaged dots of all the recording cells and the gray lines are standard error dots of them); the histogram on the right of B is the Kv1.3 current density at +40 mV spot. **(C)** Recording at same protocol on the Treg cells of CHF patients, the elevation of the current density (∼2.5-fold of the healthy volunteers, *n* = 22 vs. 20, ^∗∗^*P* < 0.01) was obtained at +40 mV of voltage clamp. **(D)** Relative level of mRNA and protein expressions of Kv1.3 channels of Tregs of the CHF patients were also elevated significantly compared to the control (*n* = 10 vs. 3 for mRNA and *n* = 6 vs. 6 for protein, ^∗∗^*P* < 0.01). All the above graphs elucidated that during the CHF states, Tregs were activated and secreted cytokines, i.e., IL-10 and TGF-β, though the proliferation of Tregs was suppressed significantly (according to the results of **Figure 1**).

ELISA assays showed IL-10 and TGF-β level in CHF patients plasma were both significantly elevated compared to the normal control (^∗∗^*P* < 0.01) (IL-10: 36.84 ± 5.42 vs. 22.21 ± 3.26 pg/mL, *n* = 30; TGF-β: 5131.25 ± 829.65 vs. 1775.83 ± 541.16 pg/mL, *n* = 30) (**Figure 2A**). Noteworthily, the elevation of IL-10 was significantly lower than the elevation of TGF-β (^∗∗^*P* < 0.01).

#### Kv1.3 Current of Treg Cell Is Elevated in the CHF Patients

By whole-cell patch clamp, treatment with 3 nM Psora-4, a Kv1.3 channel-sensitive inhibitor, was shown to suppress the *I–V* curve significantly with the ramp recording; at +40 mV. The current density was (106.11 ± 10.47) pA/pF at average for the control vs. (35.6 ± 3.51) pA/pF for the Psora-4 perfusion (*n* = 15, ^∗∗^*P* < 0.01) (**Figure 2B**), indicating that the monitored line was indeed the Kv1.3 current.

The average Kv1.3 current density curve elicited from Tregs of CHF patients was significantly higher than that of Tregs from the normal control. At +40 mV in particular, the current density in the CHF patients was ∼2.4-fold higher than in the control group (259.57 ± 19.07 vs. 107.09 ± 9.74 pA/pF, *n* = 22 vs. 20, ^∗∗^*P* < 0.01) (**Figure 2C**). The capacitance levels in control and CHF rat Treg cells were similar (1.11 ± 0.02 vs. 1.14 ± 0.01 pF in CHF vs. control, *P* > 0.05, not shown in the figure).

The relative expressions of mRNA and protein of Kv1.3 channels also risen remarkably in CHF patients compared to normal control (mRNA: 2.74 ± 0.57 vs. 1.00 ± 0.06, *n* = 10 vs. 6, ^∗∗^*P* < 0.01; protein: 0.27 ± 0.01 vs. 0.14 ± 0.01, *n* = 6 vs. 6, ^∗∗^*P* < 0.01) (**Figure 2D**).

### Immune System Alterations in CHF Model Rats

#### Plasma Levels of Inflammatory Factors Are Significantly Elevated in CHF

**FIGURE 3:**
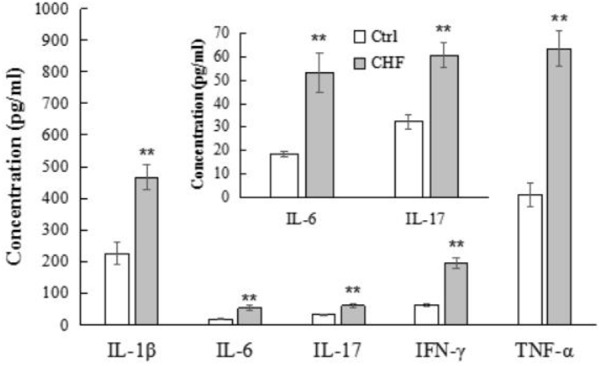
The histograms comparison of plasma levels of the inflammatory cytokines including IL-1β, IL-6, IL-17, IFN-γ, and TNF-α of control vs. CHF rats (determined by ELISA). All the inflammatory cytokines levels in CHF group were significantly higher than that in the control group (*n* = 12, ^∗∗^*P* < 0.01). The inner histogram comparison graph of IL-6 and IL-17 levels is zoomed in from the bigger one since their levels were relatively lower than other three cytokines.

The plasma levels of IL-1β, IL-6, IL-17, IFN-γ, and TNF-α in the CHF group were obviously higher than that in the control group (*n* = 12, ^∗∗^*P* < 0.01), as determined by ELISA (**Figure 3**).

#### Eplerenone Suppresses the Kv1.3 Current, Which Is Elevated in the CHF Model

**FIGURE 4:**
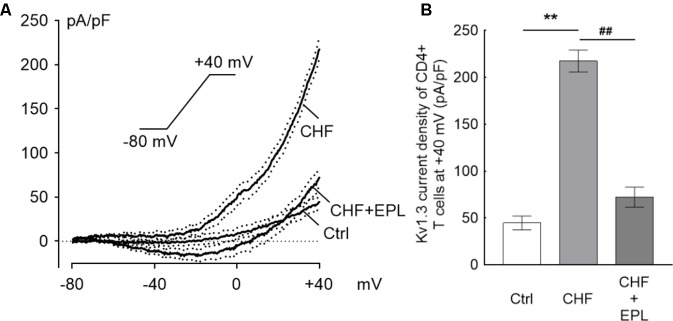
Eplerenone suppresses the Kv1.3 current, which is elevated in the CHF model. The ramp line was elicited with whole-cell patch clamp mode. The ramp protocol stimulation is sustaining from –80 mV to +40 mV of 300 ms duration applied every 30 s. **(A)** The original current density trace elicited from Tregs of the CHF rats was significantly higher than that of Tregs from the control rats (*n* = 10 cells for each group); 30 μM eplerenone (EPL) markedly suppressed the ramp line of the Kv1.3 current density in the CHF model rats (the black traces are averaged dots of the current density and the dotted lines are standard error bars). **(B)** At +40 mV in particular, the current density was ∼5-fold higher in the CHF rats than in the control rats (^∗∗^*P* < 0.01); the suppression of the current density by EPL was calculated as ∼67% comparing to the CHF rats (^##^*P* < 0.01).

The original average Kv1.3 current density trace elicited from Tregs of the CHF rats was significantly higher than that of control rats, and 30 μM EPL furthermore markedly suppressed the ramp trace of the Kv1.3 current density in the CHF model rats (*n* = 10) (**Figure 4A**). At +40 mV in particular, the current density was ∼5-fold higher in the CHF rats than that in the control rats (44.63 ± 7.43 vs. 217.34 ± 11.74 pA/pF in control vs. CHF, ^∗∗^*P* < 0.01); the suppression of the current density by EPL was calculated as ∼67% (72.13 ± 10.73 pA/pF, *n* = 10, ^##^*P* < 0.01) (**Figure 4B**). The average capacitance value of Treg cells in the three groups were similar (1.38 ± 0.19, 1.23 ± 0.13, and 1.21 ± 0.12 pF in control, CHF and CHF + EPL, *n* = 10, *P* > 0.05, not shown in the figure).

### Co-incubation of CFs With Tregs

#### Inhibition of Tregs and CF Proliferation by Eplerenone

**FIGURE 5:**
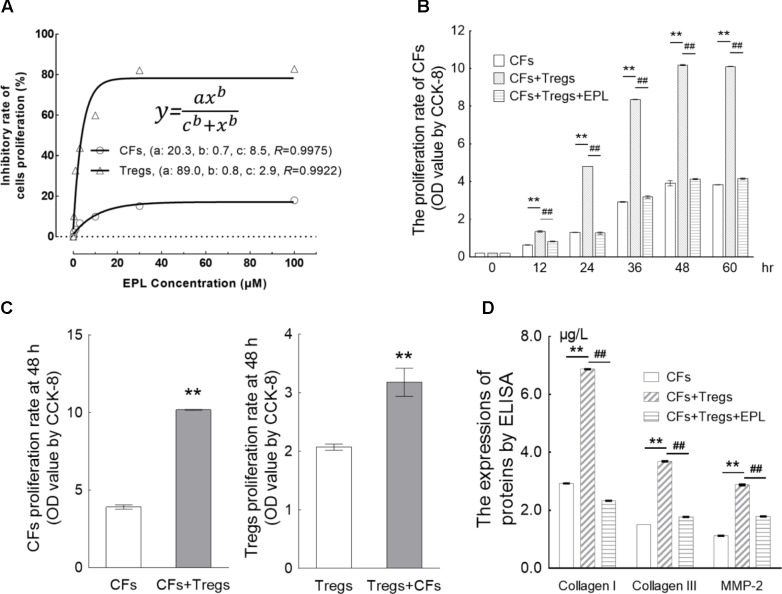
Eplerenone suppresses the proliferation of CFs induced by co-incubation with Tregs. **(A)** After 48-h treatment of Tregs and CFs with eplerenone at 0.1, 0.3, 1, 3, 10, 30, and 100 μM, cell viability (by CCK-8 assay, *n* = 8) of both cell types was inhibited in a concentration-dependent manner in accordance with the Hill equation: $y=\frac{{ax}^{b}}{c^{b}+x^{b}}$. The IC_50_s for Tregs and CFs were calculated as 2.9 and 8.5 μM, respectively. 30 μM eplerenone suppressed cell viability by 82.77 and 17.94% in Tregs and CFs, respectively, after which the viabilities stabilized. Accordingly, 30 μM was chosen as the eplerenone dose for subsequent experiments. **(B)** Tregs induced the proliferation of CFs in a time-dependent manner (CFs + Tregs vs. CFs, *n* = 8, ^∗∗^*P* < 0.01 every 12 h). The percentage of proliferation in the CFs + Tregs group was ∼2.6-fold higher than that in the CFs group at 48 h (maximum proliferation was achieved with Tregs induction). 30 μM eplerenone suppressed the augmented proliferation rate at every time point (^##^*P* < 0.01), even down to the levels of CF proliferation in the absence of Tregs. **(C)** After 48 h of co-culture, proliferation of the CFs + Tregs cultures was significantly higher than that of the single cultures: OD values of CFs vs. CFs + Tregs were 3.91 ± 0.14 vs. 10.18 ± 0.03, while those of Tregs vs. Tregs + CFs were 2.07 ± 0.15 vs. 3.18 ± 0.68 (*n* = 8, both ^∗∗^*P* < 0.01). **(D)** After 48-h of CFs + Tregs co-culture, the expression levels of collagen I, collagen III, and MMP-2 (by ELISA assay) were elevated by 2.34-, 2.46-, and 2.57-fold relative to the levels in the CFs cultured alone (CFs + Tregs vs. CFs, *n* = 8, ^∗∗^*P* < 0.01), whereas they were suppressed to 33.92, 47.97, and 62.15% of these elevated levels by 30 μM eplerenone treatment (CFs + Tregs + EPL vs. CFs + Tregs, *n* = 8, ^##^*P* < 0.01).

After 48-h treatment of Tregs and CFs with eplerenone at 0.1, 0.3, 1, 3, 10, 30, and 100 μM (*n* = 8) (**Figure 5A**), cell viability of both cell types was inhibited in a concentration-dependent manner in accordance with the Hill equation: $y=\frac{{ax}^{b}}{c^{b}+x^{b}}$. The IC_50_s for Tregs and CFs were calculated as 2.9 and 8.5 μM, respectively. At concentration of 30 μM, eplerenone suppressed cell viability by 82.77 and 17.94% in Tregs and CFs, respectively, after which the viabilities stabilized. Accordingly, 30 μM was chosen as the eplerenone dose for subsequent experiments.

#### Eplerenone Suppresses the Proliferation of CFs Stimulated by Co-incubation With Tregs

Tregs were shown to induce the proliferation of CFs in a time-dependent manner (CFs + Tregs vs. CFs, *n* = 8, *P* < 0.001 every 12 h) (**Table 2** and **Figure 5B**). The rate of proliferation in the CFs + Tregs group was ∼2.6-fold higher than that in the CFs group at 48 h (maximum proliferation was achieved with Tregs induction). Treatment with 30 μM eplerenone was shown to suppress the augmented proliferation rate at every time point (*n* = 8, *P* < 0.001), even down to the levels of CF proliferation in the absence of Tregs. After 48 h of co-culture, proliferation of the CFs + Tregs cultures was significantly higher than that of the single cultures: OD values of CFs vs. CFs + Tregs were 3.91 ± 0.14 vs. 10.18 ± 0.03, while those of Tregs vs. Tregs + CFs were 2.07 ± 0.15 vs. 3.18 ± 0.68 (*n* = 8, both *P* < 0.001) (**Figure 5C**).

**Table 2:** The proliferation of CFs over the time curve stimulated by Tregs in co-incubation by CCK-8 assay ($\overset{¯}{x}$ ± s, *n* = 8).

	OD value					
	
	0 h	12 h	24 h	36 h	48 h	60 h
CFs	0.19 ± 0.00	0.63 ± 0.01	1.30 ± 0.01	2.92 ± 0.02	3.91 ± 0.14	3.83 ± 0.01
CFs + Tregs	0.19 ± 0.00	1.35 ± 0.04^∗∗^	4.81 ± 0.00^∗∗^	8.36 ± 0.01^∗∗^	10.18 ± 0.03^∗∗^	10.11 ± 0.01^∗∗^
CFs + Tregs + EPL	0.19 ± 0.00	0.82 ± 0.02^##^	1.26 ± 0.06^##^	3.18 ± 0.07^##^	4.13 ± 0.03^##^	4.15 ± 0.04^##^


**^∗∗^P < 0.01 for CFs + Tregs vs. CFs, ^##^P < 0.01 for CFs + Tregs + EPL vs. CFs + Tregs*.*

#### Eplerenone Suppresses ECM Secretion From CFs Co-incubated With Tregs

Collagen I, collagen III, and MMP-2 are typical extracellular matrix (ECM) factors in the CF cultures, and were thus used to further evaluate CF proliferation. After 48h of CFs + Tregs co-culture, the expression levels of collagen I, collagen III, and MMP-2 were elevated by 2.34-, 2.46-, and 2.57-fold relative to the levels in the CFs cultured alone (CFs + Tregs vs. CFs, *n* = 8, *P* < 0.001), whereas they were suppressed to 33.92, 47.97, and 62.15% of these elevated levels by 30 μM eplerenone treatment (CFs + Tregs + EPL vs. CFs + Tregs, *n* = 8, *P* < 0.001) (**Figure 5D**).

#### Eplerenone Primarily Antagonizes the Augmentation of Intracellular TGF-β in Tregs Co-cultured With CFs

**FIGURE 6:**
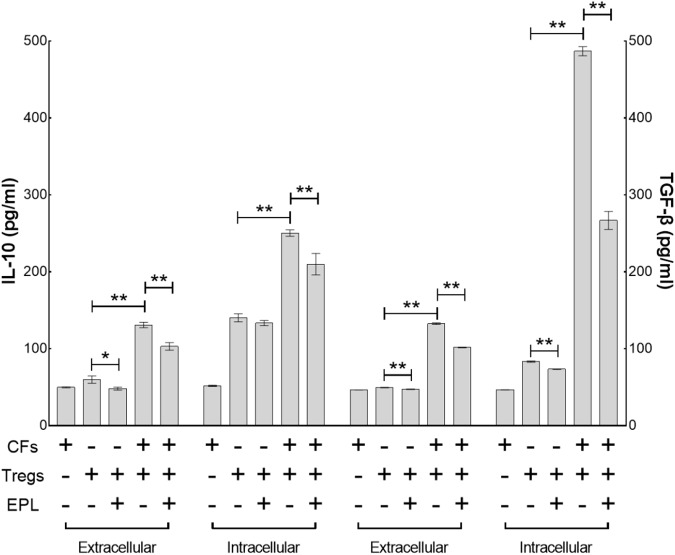
Eplerenone primarily antagonizes the augmentation of intracellular TGF-β in Tregs co-cultured with CFs. ELISA assay demonstrates that co-culture of CFs with Tregs elevated the intracellular cytokines to a significantly higher degree than it did the extracellular cytokines after 48 h (Tregs + CFs vs. Tregs, *n* = 8, *P* < 0.01). Of the intracellular cytokines, TGF-β secretion (5.8-fold from 83.21 ± 0.82 to 486.85 ± 5.95) was elevated to a greater extent than IL-10 secretion (1.8-fold from 140.24 ± 5.30 to 250.42 ± 4.17), whereas there was no significant difference between the degree of elevation for the extracellular cytokines (2.7-fold from 49.54 ± 0.33 to 132.52 ± 1.08 for TGF-β and 2.2-fold from 59.98 ± 4.78 to 130.82 ± 3.54 for IL-10). Treatment with 30 μM eplerenone was shown to suppress intracellular TGF-β levels (45.2% suppression from 486.85 ± 5.95 to 266.80 ± 11.76) to a greater extent than intracellular IL-10 levels (16.2% suppression 250.42 ± 4.17 to 209.91 ± 14.01), whereas the extent of suppression of the extracellular cytokines levels by eplerenone did not differ significantly (23.3% suppression from 132.52 ± 1.08 to 101.60 ± 0.41 for TGF-β and 21.2% suppression from 130.82 ± 3.54 to 103.02 ± 4.99 for IL-10) (^∗^*p* < 0.05, ^∗∗^*p* < 0.01).

Tregs primarily secrete TGF-β and IL-10 upon activation and/or proliferation. Co-culture of CFs with Tregs elevated the intracellular cytokines to a significantly higher degree than it did the extracellular cytokines after 48 h (Tregs + CFs vs. Tregs, *n* = 8, *P* < 0.01) (**Figure 6**). Of the intracellular cytokines, TGF-β secretion (5.8-fold from 83.21 ± 0.82 to 486.85 ± 5.95) was elevated to a greater extent than IL-10 secretion (1.8-fold from 140.24 ± 5.30 to 250.42 ± 4.17), whereas there was no significant difference between the degree of elevation for the extracellular cytokines (2.7-fold from 49.54 ± 0.33 to 132.52 ± 1.08 for TGF-β and 2.2-fold from 59.98 ± 4.78 to 130.82 ± 3.54 for IL-10). Treatment with 30 μM eplerenone was shown to suppress intracellular TGF-β levels (45.2% suppression from 486.85 ± 5.95 to 266.80 ± 11.76) to a greater extent than intracellular IL-10 levels (16.2% suppression 250.42 ± 4.17 to 209.91 ± 14.01), whereas the extent of suppression of the extracellular cytokines levels by eplerenone did not differ significantly (23.3% suppression from 132.52 ± 1.08 to 101.60 ± 0.41 for TGF-β and 21.2% suppression from 130.82 ± 3.54 to 103.02 ± 4.99 for IL-10).

#### Eplerenone Antagonizes the Augmentation of Kv1.3, KCa3.1, and CRAC Channel mRNA Expression by Tregs Co-cultured With CFs

**FIGURE 7:**
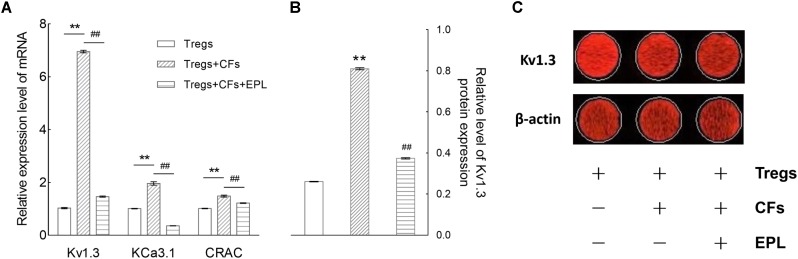
Eplerenone antagonizes the augmentation of mRNA and protein expression of Kv1.3 channel (and/or other channels) by Tregs co-cultured with CFs. **(A)** After 48 h of co-culture, the mRNA expression levels (by RT-qPCR) of Kv1.3, KCa3.1, and CRAC (Ca^2+^ release-activated Ca^2+^) channels were enhanced by 6.76-, 1.94-, and 1.46- fold, respectively (Tregs + CFs vs. Tregs, *n* = 8, ^∗∗^*P* < 0.01), whereas treatment with 30 μM eplerenone suppressed these levels by 78.98, 81.68, and 17.93%, respectively (Tregs + CFs + EPL vs. Tregs + CFs, *n* = 8, ^##^*P* < 0.01). Accordingly, the protein expression of Kv1.3 channels were detected shown in **(B)**. The relative levels of Kv1.3 channel protein expression in the Tregs + CFs group were increased by 2.11-fold relative to the Tregs group (*n* = 8, ^∗∗^*P* < 0.01), and 30 μM eplerenone suppressed these elevated protein expression levels by 52.85% (Tregs + CFs + EPL vs. Tregs + CFs, *n* = 8, ^##^*P* < 0.01). **(C)** Original images of Kv1.3 channel protein detecting by In-Cell Western Blotting.

After 48 h of co-culture, the mRNA expression levels of Kv1.3, KCa3.1, and CRAC (Ca^2+^ release-activated Ca^2+^) channels were elevated by 6.76-, 1.94-, and 1.46-fold, respectively (Tregs + CFs vs. Tregs, *n* = 8, *P* < 0.01), whereas treatment with 30 μM eplerenone suppressed these levels by 78.98, 81.68, and 17.93%, respectively (Tregs + CFs + EPL vs. Tregs + CFs, *n* = 8, *P* < 0.01) (**Figure 7A**).

#### Eplerenone Antagonizes the Augmentation of Kv1.3 Channel Protein Expression by Tregs Co-cultured With CFs

The relative levels of Kv1.3 channel protein expression in the Tregs + CFs group were elevated by 2.11-fold relative to the Tregs group (*n* = 8, *P* < 0.01), and 30 μM eplerenone suppressed these elevated protein expression levels by 53.85% (Tregs + CFs + EPL vs. Tregs + CFs, *n* = 8, *P* < 0.01) (**Figures 7B,C**).

#### Eplerenone Has High Affinity With Kv1.3 Channels Protein

**FIGURE 8:**
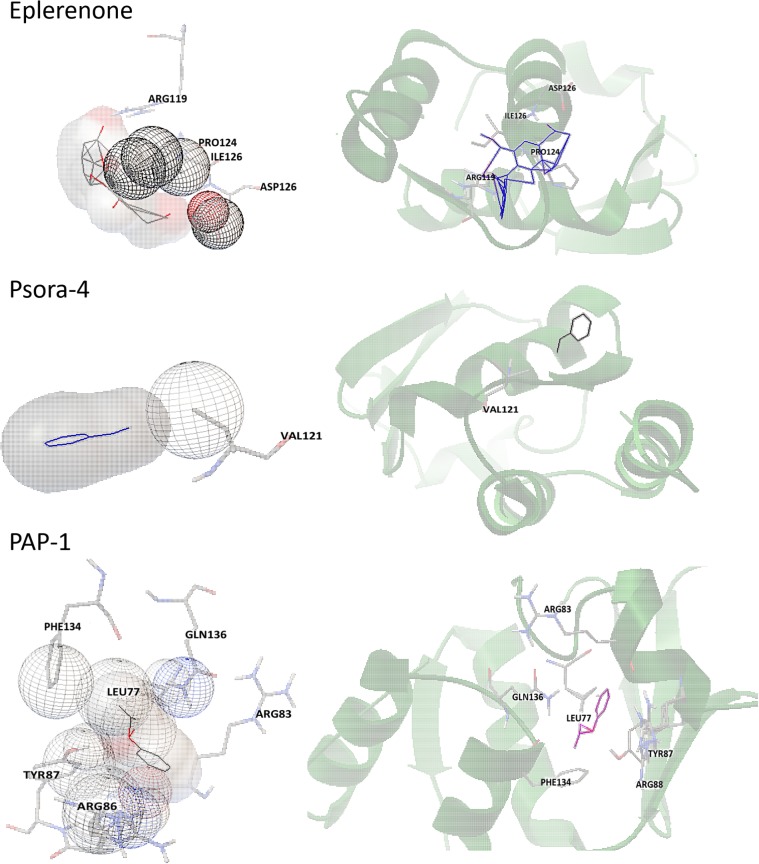
Stereo view of important residues in Kv1.3. Protein is shown in transparent cartoon, ligands and important residues in sticks.

Comparing to the known selective Kv1.3 channels blockers (PAP-1 and Psora-4), the binding energy between Eplerenone and Kv1.3 channels is higher. Though with less binding site, the H bonds makes the binding more stabilized (**Table 3** and **Figure 8**).

**Table 3:** The scores of docking and the residues.

	Binding Energy	H Bonds	Other residues
Eplerenone	-7.25	ARG119	PRO124; ILE125; ASP126
PAP-1	-4.98		ARG83; ARG88; TYR87; PHE134; LEU77; GLN136
Psora-4	-4.31		VAL121


## Discussion

Extensive evidence demonstrates that immune activation and inflammation are the important pathophysiological mechanisms involved in heart failure. Neurohormonal activation is considered an important factor in causing inflammation and development of heart failure. It is well known that aldosterone can cause alterations in the cardiovascular system, such as fibrosis, cardiac remodeling and hypertrophy. Recently, many clinical trials confirmed that aldosterone is an independent pro-inflammatory factor in key organs (Gilbert and Brown, 2010; Brown, 2013) and activates the immune system by producing inflammatory mediators in the heart, endothelium, fibroblasts, and circulating cells (Kasal and Schiffrin, 2012) to promote the progress of heart failure.

T helper lymphocytes (Th) is the main effector cell type that mediates immune response, and a Th17/Treg imbalance is the manifestation of a series of inflammatory responses. Tregs make a difference in regulating the immune response. It was shown that the quantity of Th17 cell is elevated in the total blood of patients with CHF, whereas the quantity of Treg cell is reduced (Li et al., 2010); however, another clinical trial demonstrated that Th17 cells in total blood don’t grow in patients with heart failure (Zhu et al., 2012), possibly due to a decay in Tregs and an increase in Th17/Treg ratio (Friedel and Todd, 1988). Therefore, the function of Treg cells in heart failure may be more significant than previously thought. A few studies and reviews reported that Tregs are the target in the protection of myocardial and vascular organ damage and remodeling process (Gilbert and Brown, 2010; Brown, 2013; Ramos et al., 2016). It’s also has been hinted that intravenous Tregs can reverse myocardial fibrosis mediated by the secretion of IL-10 (Cao et al., 2013). In this study, patients with CHF showed markedly imbalance of Th17/Tregs ratio with significantly higher Th17 cells and lower Treg cells compared to the healthy volunteers. Furthermore, although the serum level of both IL-10 and TGF-β elevated significantly, the elevation of TGF-β was higher than that of IL-10. As we know, TGF-β is primarily secreted from Tregs but IL-10 is not. Therefore, our results suggest the proliferation of Tregs might not parallel the activation of Tregs.

In the context of the signaling pathway of T cell activation, it is acknowledged that Kv1.3 channels maintain the resting potential of T lymphocytes and enable T cells to be activated. When T lymphocytes are activated under inflammatory conditions, the KCa3.1 channels drive membrane potential downward to hyperpolarization, thereby promoting CRAC channels open to facilitate Ca^2+^ influx, and the resulting Ca^2+^ elevation initiates the transcription of various cytokines via calcium-dependent protein kinase to promote efficient immunity. The motility of Kv1.3 channels can be used as a marker of functional viability of T cells (Lopez-Sendon, 2013) and a therapeutic target in the prevention of immune diseases. Hence, the function of Kv1.3 channels on Tregs was detected by whole-cell patch clamp technique to evaluate the activation of Tregs. The Kv1.3 channel current density in the CHF patients was significantly higher than the healthy people. And the same increasing tendencies were found in the mRNA and protein expressions of Kv1.3 channels.

In this study, coronary artery ligation was used to establish a myocardial infarction model and animals were further subjected to exhausted swimming to accelerate CHF development. The model inclusion rate was ∼50%. Cardiac function and structure were assessed by catheterization, ultrasound, Masson staining, and BNP level measurements, all of which confirmed that the inclusive CHF model was consistent with the expected clinical indicators (see **Appendix**).

In the CHF model established in this study, IL-1β, 6, 17, IFN-γ, and TNF-α levels were all elevated, as expected. IL-1β, IFN-γ, and TNF-α are involved in almost all inflammatory processes. IL-6 participates in autoimmune diseases such as hepatitis and multiple sclerosis, the secretion of which could be induced by TNF-α. IL-17, only secreted by Th17 cells, acts as a unique inflammatory factor in chronic inflammatory diseases. In the present model of CHF, all the key cytokines were shown to highly express. The 10-week CHF model rats likely still sustained the inflammatory status, and elevation of both IL-6 and IL-17 may therefore have facilitated the differentiation of CD4^+^ T lymphocytes to Th17 cells.

In the CHF model rats of this study, the Kv1.3 current density of Tregs was ∼5-fold higher than that of Tregs in the control rats. Consistent with the corresponding *in vitro* results of patients, it seems that Tregs were also activated in the CHF model, and 30 μM eplerenone could suppressed the current density in CHF model rats by ∼66.8%, which surprised us that a selective aldosterone inhibitor might directly inhibit Kv1.3 channels function since the suppressant alteration occurred within short period of time (∼15 min). In addition, docking calculation confirmed eplerenone has a higher affinity even than the selective Kv1.3 channel blockers, which suggests a direct action of eplerenone on Kv1.3 channel. To further confirm it with direct evidence, we need an appropriate cell line which possesses more Kv1.3 channels but without aldosterone receptors. Unfortunately, no such cell line was found until now.

To explain whether Tregs induce cardiac fibrosis or reverse cardiac fibrosis, we conducted the co-incubation of Tregs and cardiac fibroblasts. The features of two cells are different, Tregs are suspension cells, while cardiac fibroblasts are adherent cells, so after co-incubation the two cells could be easier to separate and be accurately detected with relative indicators. The discussion as following:

In the CFs and Tregs co-culture system used in this study, the proliferation rates of both cell types were significantly induced by each other; however, CF proliferation was induced by Tregs to a greater extent than Tregs by CF (160.6% vs. 53.4% proliferation rates). After 48 h of co-culture of CFs with Tregs, extracellular TGF-β levels were shown to elevate to a greater extent than that of IL-10, and the fold change of intracellular TGF-β augmentation (5.8) was significantly higher than that of IL-10 (1.8). Therefore, the proliferation of CFs induced by Tregs is assumed to be predominantly caused by TGF-β secretion.

Although TGF-β and IL-10 secretion was little suppressed by eplerenone treatment in Tregs cultured alone, intracellular TGF-β (45.2% diminished) and lesser degree of intracellular IL-10 (16.2% diminished) secretion was significantly suppressed by eplerenone treatment in Tregs co-incubated with CFs. The degree of suppression of extracellular TGF-β (23.3% diminished) was comparative to that of extracellular IL-10 (21.2% diminished). The proposed mechanism for the observed suppression is that eplerenone antagonizes adoptive Tregs (proliferated Tregs by autocrine secretion) rather than natural ones through inhibition of Kv1.3 channels, which potentially act as markers or targets of activation and/or proliferation of adoptive Tregs.

In the 48 h co-culture system described here, the expression of Treg channels were all elevated, furthermore, the Kv1.3 channels were most significantly elevated (almost sevenfold for mRNA and threefold for protein alterations).

The effect of eplerenone on the proliferation of CFs and Tregs indicated a dose-dependent inhibition. At 30 μM, eplerenone markedly suppressed Tregs proliferation; significantly down- regulated the augmented secretion of ECMs from CFs induced by co-incubation of Tregs.

## Conclusion

**FIGURE 9:**
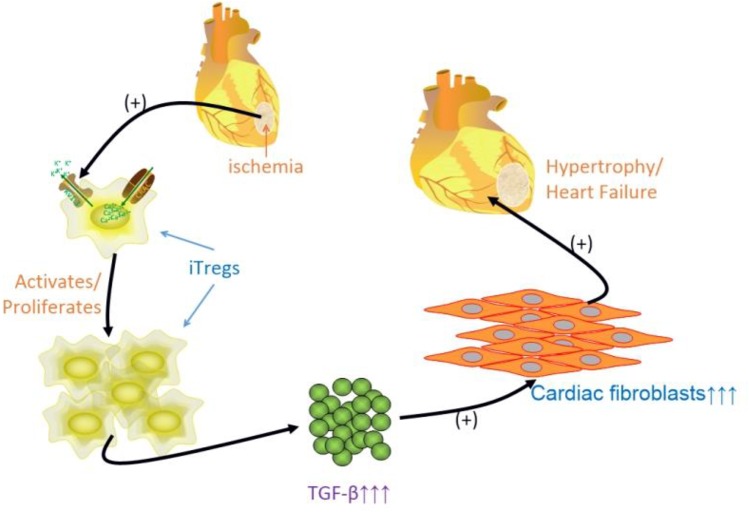
Schematic graph of the idea of the article. Some inflammatory states of heart disease, i.e., ischemia, atherosclerosis, myocardial infarction, late stage of CHF, and etc., activate immuno-response, induced regulatory T lymphocytes (iTregs) is activated/proliferated by activating Kv1.3 channels to assist Ca^2+^ influx, and releasing much more TGF-β (less IL-10 relatively), which multiplies cardiac fibroblasts leading to cardiac fibrosis to process the hypertrophy, even to heart failure.

Late-stage CHF is characterized by constant activation of the immune system, in which adoptive Tregs (induced Tregs, iTregs) are activated and/or induced to proliferate by Kv1.3 channel activation, subsequently through autocrine secretion or paracrine secretion of predominantly TGF-β and to a much lesser extent IL-10 to stimulate cardiac fibroblast proliferation (cardiac fibrosis), thereby facilitating the progression to heart failure (**Figure 9**). The anti-inflammatory effect of eplerenone on the CHF process may occur via antagonization of Kv1.3 channels in Tregs, suppression of Tregs activation/proliferation. Therefore, eplerenone could play an alleviation role to cardiac fibrosis during the development of CHF.

## Ethics Statement

This study was carried out in accordance with the recommendations of Measures for the Ethical Review of Biomedical Research Involving Human Subjects, China National Standardization Administration Committee. The protocol was approved by the Ethics Committee of First Affiliated Hospital of Xinjiang Medical University. All subjects gave written informed consent in accordance with the Declaration of Helsinki. This study was carried out in accordance with the recommendations of Laboratory Animals—Guideline of Welfare and Ethics, China National Standardization Administration Committee. The protocol was approved by the Ethics Committee of First Affiliated Hospital of Xinjiang Medical University.

## Author Contributions

All persons who have made substantial contributions to the work reported in the manuscript. The listed authors agree to be accountable for all aspects of the work in ensuring that questions related to the accuracy or integrity of any part of the work are appropriately investigated and resolved. And individual contributes to the manuscript cover as follows: L-FC, QX, and BZ conception or design of the work. P-PS, C-JL, BZ, S-HL and YW data collection. L-FC, QX, and BZ data analysis and interpretation. L-FC drafted the article. QX, BZ, and ZS critical revision of the article. P-PS, C-JL, QX, BZ, S-HL, YW, ZS, and L-FC final approval of the version to be published.

## Conflict of Interest Statement

The authors declare that the research was conducted in the absence of any commercial or financial relationships that could be construed as a potential conflict of interest.

## Figures and Tables

**FIGURE 10:**
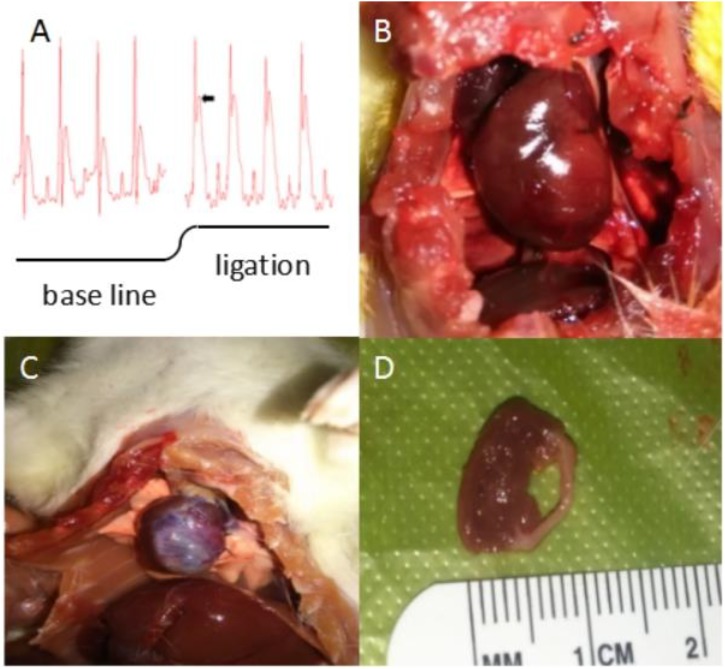
Physical features of rat hearts after coronary artery ligation (CAL). **(A)** Classic significant S-T segment elevation (ΔS-T segment > 0.1 mV is the criteria for myocardial infarction) by ECG appeared immediately when the left anterior descending coronary artery was ligated. **(B)** Ischemic pale-turning at the local precordia was visible to the unaided eye after a few minutes of CAL. **(C)** After 10 weeks, the rat hearts exhibited different degrees of fibrosis. Large-scale cardiac fibrosis of praecordia was distinctly observed, even spreading to apex of the heart and the whole heart shrunk to smaller. **(D)** Dissection of the hearts into cross-sections revealed that the ventricular wall of the left heart was thinning, and myocardial tissue was replaced by fibrosis.

**FIGURE 11:**
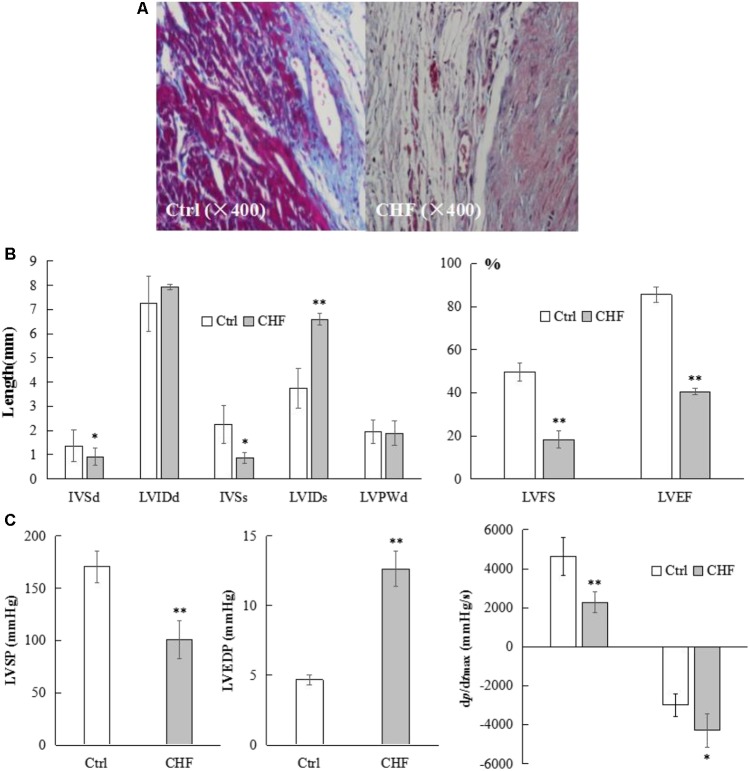
Microscopic morphological, general structural and functional alterations in the heart of CHF model rats. **(A)** Microscopic view (400×) of representative myocytes in control and CHF by Masson staining (*n* = 10). Most myocytes in the control exhibited relatively clean and neatly ordered muscular stripes (red dyed) with less collagen fibers (blue dyed) and nuclei, whereas the CHF myocytes exhibited more messy, unordered, cord-like collagen fibers instead of stripes, where some collagen fibers were found to be wrapped together in masses, forming flakes. In the CHF rats, very few complete muscular fibers were identified under high magnification. **(B)** The histograms comparison of cardiac structure parameters in control and CHF rats (*n* = 25) measured by Doppler ultrasound. The IVSd and IVSs in the CHF group were thinner than those in the control group (^∗^*P* < 0.05), while the LVIDs was significantly thicker in the CHF group than in the control group (^∗∗^*P* < 0.01). The LVIDd did not differ between the two groups. The LVFS and LVEF values were lower in the rats with CHF than in the control rats (^∗∗^*P* < 0.01). **(C)** The histograms comparison of hemodynamic parameters in control and CHF rats (*n* = 17) by right carotid artery catheterization into the left ventricle. LVSP, +d*p*/d*t*_max_ and –d*p*/d*t*_max_ in CHF group rats decreased significantly (^∗^*P* < 0.05, ^∗∗^*P* < 0.01), whereas LVEDP in CHF group rats increased significantly (^∗∗^*P* < 0.01), compared with the control group rats.

## Footnotes

1 https://pubchem.ncbi.nlm.nih.gov/

## APPENDIX

### Rat Model of CHF: Confirmation

#### Physical Features of Rat Hearts After Coronary Artery Ligation

Following successful ligation of the left anterior descending coronary artery in rats, ischemic S-T segment elevation was determined by ECG, ischemic pale-turning at the local precordia was visible to the unaided eye, and a decreased heart rate and a weak heartbeat were observed. After 10 weeks, the rat hearts exhibited different degrees of fibrosis. Dissection of the hearts into cross-sections revealed that the ventricular wall of the left heart was thinning, and myocardial tissue was replaced by fibrosis (**Figure 10**).

#### Microscopic Morphological Examination of Hearts in CHF Status

Approximately 30% of the rats established for CHF model died, where death occurred mainly during the 1st day after ligation (∼5% died during swimming). Microscopic assessment (*n* = 10) of cardiac myocytes by Masson staining and the use of different magnifications revealed that most myocytes in the control animals exhibited relatively clean and neatly ordered muscular stripes (red dyed) with less collagen fibers (blue dyed) and nuclei, whereas the CHF myocytes exhibited more messy, unordered, cord-like collagen fibers instead of stripes, where some collagen fibers were found to be wrapped together in masses, forming flakes. In the CHF rats, very few complete muscular fibers were identified under high magnification (**Figure 11A**).

#### Cardiac Structure Parameters in CHF Measured by Doppler Ultrasound

The IVSd and IVSs in the CHF group were thinner than those in the control group (*n* = 25, ^∗^*P* < 0.05), while the LVIDs was significantly thicker in the CHF group than in the control group (*n* = 25, ^∗∗^*P* < 0.01). The LVIDd did not differ between the two groups (*n* = 25). The LVFS and LVEF values were lower in the rats with CHF than in the control rats (*n* = 25, ^∗∗^*P* < 0.01) (**Figure 11B**).

#### Hemodynamic Parameters During Heart Failure by Right Carotid Artery Catheterization Into the Left Ventricle

Compared with the corresponding parameters in the control group, the left ventricular systolic pressure (LVSP) and the maximum rate of rise/drop of left ventricular pressure (±d*p*/d*t*_max_) in the rats with CHF decreased (*n* = 17, ^∗^*P* < 0.05, ^∗∗^*P* < 0.01), whereas the left ventricular end-dilating pressure (LVEDP) increased (*n* = 17, ^∗∗^*P* < 0.01). There was no statistically significant difference in heart rates between the control and CHF rats (*n* = 17, 367.42 ± 18.64 vs. 372.19 ± 16.73) (**Figure 11C**).

#### Plasma BNP Levels Were Significantly Elevated in CHF Rats

Plasma BNP levels in the control and CHF groups were 378.54 ± 13.72 and 572.63 ± 21.86 ng/mL, respectively, demonstrating a significant increase (*n* = 19, ^∗∗^*P* < 0.01) 6 weeks after the ligation of the coronary artery.
